# Stimmdiagnostik bei phonochirurgischen Eingriffen

**DOI:** 10.1007/s00106-021-01053-w

**Published:** 2021-05-31

**Authors:** Berit Schneider-Stickler

**Affiliations:** Klinische Abteilung Phoniatrie-Logopädie, Univ.-HNO-Klinik, Medizinische Universität Wien, Währinger Gürtel 18–20, 1090 Wien, Österreich

**Keywords:** Diagnostische Techniken und Verfahren, Diagnostische Selbstbeurteilung, Endoskopie, Stimmqualität, Symptombeurteilung, Phonochirurgie, Diagnostic techniques and procedures, Diagnostic self-evaluation, Endoscopy, Voice quality, Symptom assessment, Phonosurgery

## Abstract

Stimmdiagnostische Untersuchungen vor und nach phonochirurgischen Eingriffen dienen zum einen der detaillierten Planung der chirurgischen Vorgehensweise und zum anderen der Nachvollziehbarkeit des postoperativen Ergebnisses. Die Stimmdiagnostik verfolgt dabei eine vielschichtige Vorgehensweise und umfasst Aspekte aus perzeptiver Stimmklangbeurteilung, Laryngostroboskopie, Stimmakustik, Aerodynamik und der Selbsteinschätzung durch die Patient*innen. Die Auswahl stimmdiagnostischer Methoden sollte sich dabei immer am aktuellen Wissensstand orientieren und das einrichtungsinterne Vorgehen prägen.

## Abstract (en)

Voice diagnostics before and after phonosurgical interventions should aim at detailed planning of surgical techniques and evaluability of postoperative outcome. They should consider multimodal concepts and include measurements of perceptual voice analysis, laryngostroboscopy, voice acoustics, aerodynamics, and self-evaluation by the patients. Selected methods for voice diagnostics should reflect current knowledge and define the departmental procedure.

Im klinischen Alltag werden alle Arten von Stimmstörungen unter dem Begriff „Dysphonien“ zusammengefasst. Hauptsymptome jeder Dysphonie sind der gestörte Stimmklang, hier in erster Linie der heisere Stimmklang, sowie Missempfindungen im Halsbereich und Einschränkungen der stimmlichen Leistungsfähigkeit. Aufgabe der Stimmdiagnostik ist es, differenzialdiagnostische Unterscheidungen zwischen organischen und funktionellen Dysphonien zu treffen und Entscheidungskriterien für das entsprechende therapeutische Vorgehen zu geben.

## Stimmdiagnostische Überlegungen

Im Hinblick auf Therapieentscheidungen einschließlich der Indikationsstellung einer phonochirurgischen Intervention zielt die Stimmdiagnostik nicht nur auf die Erkennung der zugrunde liegenden morphologischen Stimmlippenveränderungen, sondern auch auf die Beurteilung des Schweregrads der resultierenden Stimmstörung und auf die subjektive stimmliche Beeinträchtigung unter Einbeziehung möglichst standardisierter subjektiver und objektiver Kriterien. Im Rahmen phonochirurgischer Eingriffe geht es vordergründig darum, wie man durch operative Eingriffe am „Stimmapparat“ morphologische Veränderungen hinsichtlich Stimmproduktion und/oder Klangformung verbessern bzw. wiederherstellen kann.

Während audiologische Untersuchungen vor Planung einer Ohroperation unverzichtbar zum Standard gehören, werden Eingriffe an den Stimmlippen bis heute oft noch ausschließlich aufgrund morphologischer Befundkonstellationen ohne Einbeziehung stimmleistungsbeurteilender Kriterien indiziert. Reinke-Ödeme, oft Zufallsbefunde im Rahmen einer Hals-Nasen-Ohren-ärztlichen Untersuchung, werden noch immer eher zu phonochirurgischen Interventionen eingeteilt als winzige Stimmlippenrandverdickungen bei professionellen Stimmnutzern. Während ein Reinke-Ödem nicht selten bei Betroffenen kein Stimmstörungsgefühl verursacht, können feine Stimmlippenrandverdickungen, die oft nur mit hochqualitativen laryngostroboskopischen Untersuchungstechniken visualisiert werden können, bei professionellen Stimmnutzern existenzbedrohende berufliche Einschränkungen verursachen.

Phonochirurgie kann nur dann verantwortungsbewusst praktiziert werden, wenn die ausführenden Chirurg*innen die Eingriffe unter Bewertung der jeweiligen Funktionseinschränkung der Stimme detailliert beurteilen und planen. Die professionelle Analyse der menschlichen Stimme vor und nach phonochirurgischen Eingriffen sollte dabei genauso bewusst und differenziert erfolgen wie die audiologische Diagnostik vor und nach ohrchirurgischen Eingriffen. Die Planung phonochirurgischer Techniken basiert nicht nur auf exakten feinmotorischen Fähigkeiten und der Kenntnis möglicher phonochirurgischer Finessen, sondern auch auf der Kenntnis der möglichen Einflussnahme auf eines der wichtigsten menschlichen Ausdrucks- und Arbeitsmittel: der Stimme.

Für die dafür notwendigen präoperativen Untersuchungen stehen mittlerweile eine Vielzahl hochwertiger Untersuchungsmöglichkeiten und internationale Empfehlungen für standardisierte stimmdiagnostische Vorgehensweisen zur Verfügung.

Im Klinikalltag sollte ein kompakter stimmdiagnostischer Untersuchungsablauf angestrebt werden

In Anbetracht der stetig steigenden Anzahl stimmdiagnostischer Möglichkeiten, insbesondere in der wissenschaftlichen Literatur, sollte im Klinikalltag eine kompakte, praktikable und qualitätssichernde Variante eines stimmdiagnostischen Untersuchungsablaufs angestrebt werden.

Behandlungsbedarf, Behandlungszeitpunkt und Behandlungsplan ergeben sich immer aus den individuellen stimmlichen Ansprüchen der Patient*innen.

Die Entscheidung zur Phonochirurgie sollte dann getroffen werden, wenn die jeweilige Befundkonstellation eine Verbesserung bzw. Normalisierung des nachweislichen stimmlichen Defizits erwarten lässt. Die phonochirurgischen Interventionsmöglichkeiten sind je nach zugrunde liegender Pathologie vielfältig [1, 2]. Perioperative stimmdiagnostische Untersuchungen ermöglichen gezielte quantitative Vergleiche des Therapieerfolgs vor und nach phonochirurgischen Interventionen. Routinemäßige prä- und postoperative stimmdiagnostische Untersuchungen können nicht nur zur Operationsplanung und zum Nachweis von Operationserfolgen, sondern auch zum Vergleich verschiedener phonochirurgischer Vorgehensweisen und alternativer Behandlungstechniken herangezogen werden. Mit zunehmendem Anspruch der Patient*innen an ärztliche Leistungen müssen auch mögliche forensische Aspekte berücksichtigt werden. Stimmdiagnostische Untersuchungen können dabei einerseits zur Argumentation bei unrealistischen Therapiewünschen und andererseits bei eventueller patientenbezogener Unzufriedenheit zur Beweisführung des tatsächlichen Therapieerfolgs nach Phonochirurgie herangezogen werden.

## Anamneseerhebung

Die Anamnese gilt allgemein als Schlüssel zur Diagnostik von Krankheiten. Die Anamneseerhebung in der laryngologischen/phoniatrischen Ambulanz ist i. d. R. eine offene Befragung und schließt sämtliche Fragen zur Erhebung der jetzigen Anamnese, der früheren Anamnese, der Familienanamnese und der sozialen Anamnese ein. Dabei stehen Fragen zu möglichen auslösenden Ereignissen/Art/Dauer/Qualität der Stimmbeschwerden im Vordergrund. Es gilt abzuklären, ob die Stimmstörungen im Zusammenhang mit beruflicher und/oder privater Stimmbeanspruchung auftreten und ob sie diese beeinträchtigen. Stimmliche Risikofaktoren (Umgebungslärm, Rauchen, Alkohol, Begleiterkrankungen) sind gezielt zu erfragen. Immer wieder bringen Patient*innen bestehende Stimmprobleme nicht mit früheren Ereignissen, Vorerkrankungen, Dauermedikationen oder Operationen in der Vergangenheit in Zusammenhang.

Im Rahmen des Anamnesegesprächs müssen bereits absolvierte Therapien ebenso wie stimmtherapeutische Erwartungen erfragt werden.

## Standardisierte Stimmdiagnostik

Im Folgenden wird ein Überblick über die Systematik einer standardisierten Stimmdiagnostik gegeben. International kommen derzeit in der Stimmdiagnostik eine Vielzahl subjektiver, semiobjektiver und phonochirurgischer Untersuchungsmethoden zur Anwendung, die zum großen Teil auf individuellen Beurteilungskriterien beruhen und von regionalen bzw. überregionalen Einflüssen geprägt sind. Für die Diagnosestellung und Bewertung von Therapieergebnissen sind systematische und valide multimodale Untersuchungskonzepte insbesondere im Hinblick auf phonochirurgische Therapieplanungen nach einem standardisierten Protokoll notwendig.

Europäische Phoniater und Laryngologen bemühen sich seit Jahrzehnten, mit multiparametrischen Konzepten der unüberschaubaren Vielfalt an stimmdiagnostischen Untersuchungsmethoden entgegenzuwirken [3–5]. Es ist eine Aufgabe der Zukunft, diese in der täglichen Arbeit umzusetzen.

Durch die Europäische Laryngologische Gesellschaft wurde ein stimmdiagnostischer Bewertungsstandard festgelegt

Nach Eysholdt (2014) stellt die Phonochirurgie als Elektiveingriff besondere Anforderungen an eine korrekte Indikationsstellung mit entsprechender Befunddokumentation [5]. Standardmäßig sollten (Laryngo‑)Stroboskopie, Aerodynamikmessungen sowie Stimmaufnahmen und -analysen aus einem gehaltenen Ton kombiniert mit psychometrischen Fragebögen zur Selbst- und Fremdbewertung erfolgen. Durch die Europäische Laryngologische Gesellschaft (ELS) per Konsens festgelegt, sind sie seit 2001 der Bewertungsstandard, anhand dessen Indikationsstellung und Behandlungserfolg bewertet werden können. Die ELS definiert als Basisstandard folgende stimmdiagnostische Säulen [4]:

- perzeptive (Fremd‑)Beurteilung des Stimmklangs (Perzeption),
- visuelle Beurteilung der Stimmlippenschwingung (Laryngostroboskopie),
- apparative Stimmanalyse und Stimmleistungsbeurteilung (Akustik),
- Erfassung ausgewählter aerodynamischer Parameter (Aerodynamik),
- standardisierte Selbsteinschätzung der eigenen Stimme durch den Patienten (Selbstbeurteilung).

In Tab. 1 sind die Empfehlungen für die multimodale Stimmdiagnostik, basierend auf dem Basisprotokoll der ELS, mit Beispielen und Bewertungen modifiziert zusammengefasst.

**Table 1:** 

Komponente	Beispiele	Bewertung
Perzeption	Stimmklangbeurteilung nach der GRBAS-Skala oder RBH-Skala	4‑Punkte-Skala: 0 = keine Abweichung, 1 = geringgradige Störung, 2 = mittelgradige Störung, 3 = hochgradige Störung
Visuelle Beurteilung der Stimmlippenschwingung (Laryngostroboskopie)	*Beurteilung der Stimmlippenschwingungen:*	Beschreibend
	Glottisschluss	
	Regularität/Irregularität	
	Schwingungsamplituden	
	Randkantenverschieblichkeit	
	Symmetrie	
Akustische Messungen	Periodizitätsanalysen (Jitter = Frequenzvariationen, Simmer = Amplitudenvariationen)	Angabe in %
	Harmonics-to-Noise-Ratio^a^	Angaben in dB
	*Stimmfeldmessungen, z.* *B.:*	
	Höchste Frequenz	Angabe in Hz
	Geringste Intensität	Angabe in dB
	Tonhöhenumfang	Angabe in Halbtonschritten
Aerodynamische Untersuchungen	Maximale Tonhaltedauer/a:/	Angabe in s
	Phonationsquotient (Vitalkapazität/maximale Tonhaltedauer)	Angabe in ml/s
	Vitalkapazität	Angabe in ml
Subjektive Bewertung durch den Patienten	Beurteilung der Stimmqualität mithilfe stimmbezogener Fragen	Beschreibend/Scores

*GRBAS* „Grade, roughness, breathiness, asthenic, strained qualities“, d. h. Grad der Heiserkeit, Rauigkeit, Behauchtheit, Asthenie/Schwachheit, Gepresstheit/Angestrengtheit; *RBH *Rauigkeit, Behauchtheit, Heiserkeit

^a^Harmonic-to-Noise-Ratio = Verhältnis zwischen harmonischen und nichtharmonischen Geräuschanteilen des Stimmklangs

Eine standardisierte Stimmdiagnostik verfolgt das Ziel der Objektivierung und Dokumentation stimmtherapeutischer Entscheidungen und dient der Qualitätssicherung in der Medizin.

Im klinischen Alltag wird die Erhebung stimmdiagnostischer Befunde i. d. R. durch die professionelle Zusammenarbeit zwischen Laryngolog*innen/Phoniater*innen und Logopäd*innen ermöglicht. Praktisches Beispiel für ein standardisiertes interdisziplinäres Vorgehen ist das Zürcher Stimmdiagnostikprotokoll [7].

Regelmäßiger Erfahrungsaustausch, interne Supervision und wissenschaftliche Fortbildungen sichern die einrichtungsinterne Qualität der stimmdiagnostischen Befunderhebung und -interpretation.

### Perzeption

Leitsymptom einer Stimmstörung ist die Heiserkeit, die die auditiv wahrnehmbaren Abweichungen vom normalen Stimmklang zusammenfasst. Bei jedem Patient*innengespräch findet, ob bewusst oder unbewusst, eine „psychoakustische“ Beurteilung des Stimmklangs über das Ohr de*r Untersucher*in im Sinne einer auditiv-perzeptiven Stimmklangbeurteilung statt.

International haben sich 2 Bewertungssysteme durchgesetzt, zum einen die GRBAS-Skala („grade, roughness, breathiness, asthenic, strained qualities“) nach Hirano und zum anderen die davon gekürzte RBH-Skala (Rauigkeit, Behauchtheit, Heiserkeit) nach Wendler et al. [8, 9]. Beide definieren die Heiserkeit als auditives Leitsymptom einer Stimmstörung, deren Teilkomponenten zusätzlich beschrieben werden sollen (Tab. 2).

**Table 2:** 

Abk.	Englische Bezeichnung	Deutsche Bezeichnung	Beschreibung
G = H	„Grade“	Gesamtgrad der Heiserkeit	Gesamteindruck der Stimmstörung bzw. der Heiserkeit
R	„Roughness“	Rauigkeit	Störung des Stimmklangs durch den Eindruck irregulärer Schwingungsanteile, tieffrequenter Geräuschanteile oder Vocal-Fry
B	„Breathiness“	Behauchtheit	Störung des Stimmklangs durch hörbare turbulente Luftströmungsanteile, meist bedingt durch inkompletten Glottisschluss
A	„Asthenic“	Schwachheit	Auditiver Eindruck einer Stimmschwäche wie bei Hypofunktion
S	„Strained qualities“	Gepresstheit/Angestrengtheit	Auditiver Eindruck eines übermäßigen Spannungs- und Anstrengungsgrades wie bei einer Hyperfunktion

Die Parameter werden entsprechend ihrer Ausprägung auf einer 4‑Punkte-Skala graduiert:

- 0 = nicht vorhanden,
- 1 = geringgradig vorhanden,
- 2 = mittelgradig vorhanden,
- 3 = hochgradig vorhanden.

Der Gesamtgrad der Heiserkeit (G bzw. H) entspricht in der Gesamtbeurteilung nicht der Summe der anderen Faktoren, sondern der höchsten Beurteilung der Einzelfaktoren.

Der Gesamtgrad der Heiserkeit entspricht der höchsten Beurteilung der Einzelfaktoren

Die perzeptiv-auditive Stimmklangbeurteilung der Stimmklangparameter erfolgt i. d. R. für Spontansprache und für Standardtexte.

### Laryngostroboskopie

Erst die Kombination der Laryngoskopie mit einer Stroboskopie ermöglicht die visuelle Beurteilung der phonatorischen Stimmlippenmotilität und damit der Schwingungscharakteristika der Stimmlippen. Laryngostroboskopien sind sowohl starr als auch flexibel inzwischen mit exzellenter Bild- und Lichtqualität möglich. Digitale Befundaufzeichnungen sind inzwischen für Begutachtungen sowie prä- und postoperative Befundvergleiche üblicher Standard. Auch für phonochirurgische Überlegungen sind stroboskopische Aufnahmen zur Beurteilung der Schwingungseigenschaften der Stimmlippen unverzichtbar, da nicht nur der Organbefund selbst, sondern sein Einfluss auf die Stimmlippenschwingungscharakteristik ausschlaggebend für die Wahl phonochirurgischer Techniken ist. Laryngostroboskopische Untersuchungen sollten sowohl für Grundfrequenzen im Bereich der indifferenten Sprechstimmlage als auch bei hoher Phonation mit verschiedenen Intensitäten erfolgen. Die klinische Beurteilung orientiert sich im Wesentlichen an den bekannten stroboskopischen Parametern: Schwingungsamplituden, Randkantenverschieblichkeit, Schwingungssymmetrie, Irregularitäten, Phasendifferenzen und Glottisschluss [6, 10].

Die digitale Aufzeichnung der Untersuchungsbefunde ermöglicht eine spätere Befundeinsicht für Patientenbesprechungen, prä- und postoperative Vergleiche und spätere Befundauswertungen.

Seit einigen Jahren werden in einigen Stimmzentren Endoskopien mit hoher Zeitauflösung verwendet, die Hochgeschwindigkeitsendoskopie [5, 11, 12]. Mit dem Fokus auf zusätzlichen Bewegungsinformationsgewinn werden Bilddaten in Form von „Phonovibrogrammen“ komprimiert, die die beidseitigen Stimmlippenbewegungen im Seitenvergleich im Zeitverlauf objektivieren.

### Akustik

Aus einem Stimmsignal lassen sich zahlreiche Maße zur Beschreibung einer Stimme ermitteln.

Die akustische Analyse von Stimmklängen gehört in der standardisierten Stimmdiagnostik zu den unverzichtbaren Untersuchungsverfahren zur Objektivierung und Quantifizierung der akustischen Eigenschaften der Stimme. Voraussetzung dafür ist die Aufnahme und Speicherung repräsentativer Stimmsignale (Standardtexte, Standardsätze, ausgehaltene Vokale) unter standardisierten Aufnahmebedingungen (u. a. definierter Mund-Mikrofon-Abstand, Mikrofonauswahl, geräuscharme Umgebung).

In der Literatur existiert mittlerweile eine unendliche Vielzahl akustischer Stimmparameter, die jedoch oft wissenschaftlichen Fragestellungen vorbehalten sind und ein gewisses Maß an physikalisch-akustischer Expertise voraussetzen. Gelegentlich überschreiten sie jedoch das gängige praxisorientierte stimmdiagnostische Fachwissen. Einige wesentliche Parameter basierend auf Perturbationsmessungen bzw. auf spektralanalytischen Verfahren haben dennoch Eingang in die klinische Stimmdiagnostikroutine gefunden und werden bereits in einigen kommerziell erhältlichen Stimmdiagnostik-Softwareprogrammen eingebunden. Dazu zählen in erster Linie die Perturbationsparameter Jitter (mittlere Abweichung der Schwingungsfrequenz) und Shimmer (mittlere Abweichung der Schwingungsamplitude), als auch die Harmonic-to-Noise-Ratio (Verhältnis zwischen harmonischen und nichtharmonischen Geräuschanteilen des Stimmklangs/Harmonische-Geräusch-Verhältnis).

Die Stimmfeldmessung gilt als „Arbeitspferd“ zur Beurteilung der stimmlichen Leistung

Für die Beurteilung der Stimmleistung steht die Stimmfeldmessung (von einigen Autor*innen auch Stimmprofilmessung genannt) zur Verfügung. Die Stimmfeldmessung gilt als „Arbeitspferd“ zur Beurteilung der stimmlichen Leistung bzw. stimmlicher Defizite und ist in der klinischen Bedeutung vergleichbar mit der Tonaudiometrie in der Otologie. In einem x‑y-Diagramm (x = Frequenz, y = Schalldruckpegel) werden Sprechstimmleistung (in verschiedenen Steigerungsstufen von leiser Stimmgebung bis zur Rufstimme) und Singstimmleistung (Tonhöhenumfang bei leisem und lautem Singen) dokumentiert. Die Stimmfeldmessung ist eine Momentaufnahme der stimmlichen Leistung zum Untersuchungszeitpunkt, jedoch gibt sie Aufschluss über eine Vielzahl stimmlicher Beurteilungskriterien hinsichtlich Stimmkonstitution und Stimmfunktion.

Besondere Aufschlüsse bieten hierbei minimal und maximal erreichbare Intensitäten der Sing- und Sprechstimme und der Tonhöhenumfang der Singstimme. Die minimale Intensität soll dabei mit dem Ansprechen der Stimmlippenschwingungen korrelieren und Ausdruck der Geschmeidigkeit des Stimmlippenepithels und der Grundspannung der Stimmlippe sein. Fixierungen oder Versteifungen im Stimmlippenepithel bzw. im Subepithelialraum lassen Stimmlippenschwingungen erst bei höheren Intensitäten zu. Darüber hinaus lassen sich aus den Messergebnissen stimmkonstitutionelle und -konditionelle Rückschlüsse ziehen.

Derzeit inkludieren verschiedene kommerzielle Softwareprogramme die multiparametrische Auswertung der Stimmqualität mithilfe des Dysphonia-Severity-Index (DSI) nach Wuyts et al. [13]. Der DSI soll über den Grad der Dysphonie und die Stimmqualität unter Verwendung von 4 Parametern informieren:

- höchste im Stimmfeld erreichte Frequenz (F0-high in Hz),
- geringster im Stimmfeld erreichter Schalldruckpegel/Intensität (I-low in dB),
- maximale Tonhaltedauer/Phonationszeit auf /a:/ (MPT in s),
- Jitter (in %).

### Aerodynamik

Die einfachste und am leichteste durchzuführende stimmdiagnostische Messung ist die Messung der maximalen Tonhaltedauer eines mit mittlerer Intensität und auf Sprechtonhöhe ausgehaltenen Vokals /a:/.

Im Idealfall ist die zusätzliche spirometrische Bestimmung der Vitalkapazität möglich, die man zur Berechnung des Phonationsquotienten (PQ, Verhältnis aus Vitalkapazität und maximaler Tonhaltedauer) verwenden kann.

Die Berücksichtigung der Vitalkapazität im Rahmen der Stimmdiagnostik kann auf zusätzliche Lungenfunktionsprobleme hinweisen, die die Stimmleistung beeinträchtigen können, ohne dass primär ein Stimmproblem zugrunde liegt.

### Subjektive Einschätzung durch Patient*innen

Phonochirurgische Interventionen sind im Gegensatz zu beispielsweise onkologischen Operationen keine vital notwendigen Eingriffe. In der phonochirurgischen Therapieplanung haben daher die Wünsche und stimmlichen Bedürfnisse einen besonderen Stellenwert [3]. Jede phonochirurgische Intervention muss sich dem Therapiewunsch der Patient*innen unterordnen. Daher hat die präoperative Selbsteinschätzung der stimmlichen Situation und des möglichen stimmlichen Handicaps einen besonderen Stellenwert, um in der Zusammenschau von stimmdiagnostischen Befunden, phonochirurgischen Interventionsmöglichkeiten und individuellen Patient*innenansprüchen ein gemeinsames Therapiekonzept von Operateur*innen und Patient*innen zu erreichen.

Die systematisierte Selbstauskunft mithilfe von standardisierten Fragebögen ist essenziell

Fragebögen können zur Reflexion der gefühlten Stimmstörung und zur Beurteilung der Teilhabe am gesellschaftlichen Kommunikationsprozess verstanden werden und sollten erhobene klinische Untersuchungsbefunde ergänzen. Die systematisierte Selbstauskunft von Patient*innen mithilfe von standardisierten Fragebögen ist für die Diagnostik von Stimmstörungen und deren Therapie essenziell.

In Anbetracht einer inzwischen unendlichen Vielfalt stimmdiagnostischer Möglichkeiten ist es im Klinikalltag empfehlenswert, sich auf eine kompakte qualitätssichernde Minimalvariante eines stimmdiagnostischen Untersuchungsablaufs zu konzentrieren.

Nicht selten gehen minimale funktionelle oder organische Auffälligkeiten mit einem hohen Leidensdruck einher. Andere Patient*innen haben trotz ausgeprägter organischer Befunde nur geringe subjektive stimmliche Einbußen. Für die Planung phonochirurgischer Interventionen ist der Behandlungswunsch der Patient*innen anhand der stimmlichen Selbsteinschätzung maßgeblich.

Die Abfrage der subjektiven Erfahrung der Betroffenen anhand von Selbsteinschätzungsbögen spielt darüber hinaus bei vergleichenden Wirksamkeitsstudien eine große Rolle [14, 15]. Nach einem systematischen Review von Salm et al. stehen im Deutschen verschiedene Fragebögen zur Auswahl [16]:

- Classical and Modern Singing Handicap Index (CSHI/MSHI) [17],
- Stimmstörungsindex (SSI = VHI-12) [18],
- Singing Voice Handicap Index (SVHI) [19],
- Transsexual Voice Questionnaire for Male-to-Female Transsexuals (TVQMtF) [20],
- Voice Handicap Index (VHI-30) [21],
- VHI‑9 [22],
- Voice-related Quality of Life Questionnaire (V-RQOL) [23, 24].

Von diesen Fragebögen findet in der klinischen Praxis in erster Linie der Voice Handicap Index in seiner ursprünglichen Version mit 30 Items breite Anwendung. Dieser dient der Selbsteinschätzung bei Heiserkeit und/oder einem gestörten Stimmklang durch Beantwortung von 30 Fragen auf einer Bewertungsskala von 0 (nie), 1 (selten), 2 (manchmal), 3 (oft) bis 4 (immer). Kritik an diesem Fragebogen wurde immer wieder aufgrund des hohen Zeitaufwands für das Ausfüllen des Fragebogens geübt, weshalb er inzwischen in gekürzten Fassungen als SSI = VHI-12 mit 12 Fragen bzw. VHI‑9 mit 9 Fragen zur Anwendung kommt. Für die gezielte Befragung von Sänger*innen mit Stimmproblemen hat sich der mehr auf die Sprechstimme fokussierte VHI-30 (einschließlich VHI‑9 und VHI-12) nicht bewährt, sodass für die Erfassung der gesanglichen Einschränkungen der Sänger*innen speziell der SVHI als diagnostisches Selbstbeurteilungsinstrument entwickelt wurde. Wie beim VHI-30 ist die Beantwortung der Fragen auf einer Bewertungsskala mit 0 (keine Störung), 1 (leicht gestört), 2 (mittelgradig gestört) bis 3 (hochgradig gestört möglich). Zur Beurteilung des Einflusses einer Stimmstörung auf die Lebensqualität bzw. der stimmbezogenen Lebensqualität kann der V‑RQOL eingesetzt werden. Dieser besteht aus 10 Items, davon sind 4 Items sozial-emotionalen Aspekten und 6 Items physisch-funktionellen Aspekten zuzuordnen. Die Antwortkategorien reichen von 1 (kein Problem), 2 (kaum ein Problem), 3 (schon ein Problem), 4 (ein großes Problem) bis 5 (ein Problem, wie es schlimmer nicht sein könnte).

Mit den Fragebögen „Classical und Modern Singing Handicap Index“ (CSHI/MSHI) soll gezielt der Einfluss stimmlicher Beeinträchtigungen auf die Lebensqualität von Sänger*innen erhoben werden. Mit den unterschiedlichen Versionen für einerseits klassische Sänger*innen (CSHI) und andererseits nichtklassische Sänger*innen (MSHI) sollen die unterschiedlichen Anforderungen des jeweiligen Gesangsstils Berücksichtigung finden. Beide Fragebögen bestehen aus 30 Items. Jeweils 10 Items überprüfen die Auswirkungen der Probleme auf die professionelle Sing- und Sprechstimme („disability“), psychologische Auswirkungen („handicap“) und die Wahrnehmung der Qualitätsmerkmale der Singstimme („impairment“). Die Selbsteinschätzung erfolgt in der deutschen Version auf einer 4‑stufigen Skala mit 0 (nie), 1 (manchmal), 2 (oft) und 3 (immer).

Der TVQMtF ist speziell der Selbsteinschätzung von Mann-zu-Frau-Transsexuellen (Trans*Frauen) vorbehalten. In 30 Items werden verschiedene erlebte Situationen der Stimmanwendung und -akzeptanz in verschiedenen Lebenssituationen hinterfragt. Hier sind die Beantwortungen auf einer 4‑stufigen Likert-Skala mit den Stufen 1 (nie oder selten), 2 (manchmal), 3 (oft) und 4 (meist oder immer) möglich. Die Auswertung fokussiert dabei auf die Einschätzung, wie weiblich bzw. männlich die eigene Stimme wahrgenommen wird und wie weiblich bzw. männlich sie idealerweise klingen sollte.

## Fazit für die Praxis

- Stimmdiagnostische Untersuchungen vor und nach phonochirurgischen Eingriffen sind unerlässlich.
- Sie unterstützen zum einen die detaillierte Operationsplanung und dienen zum anderen der Qualitätssicherung in der Medizin.
- Fundierte stimmphysiologische und stimmakustische Kenntnisse sind Voraussetzung für eine evidenzbasierte Stimmdiagnostik.
- Die standardisierte Stimmdiagnostik verfolgt dabei eine vielschichtige Vorgehensweise und umfasst Aspekte aus perzeptiver Stimmklangbeurteilung, Laryngostroboskopie, Stimmakustik, Aerodynamik zur Stimmfunktion und Selbsteinschätzung durch Patient*innen.
- Die Auswahl stimmdiagnostischer Methoden sollte sich dabei immer am aktuellen Wissensstand orientieren und das einrichtungsinterne Vorgehen prägen.
